# Iranian Black Tea and Cowslip Extracts Induce Tumor Necrosis Factor-Alpha Secretion from Mouse Macrophage Cell Culture

**Published:** 2010

**Authors:** Mahmoud Nadi, Nariman Mosaffa, Forouzan Karimi, Mohammad Kamalinejad, Babak Farrokhi, Arash Anissian, Parviz Pakzad

**Affiliations:** a *Department of Immunology, School of Medicine, Shahid Beheshti University of Medical Sciences, Tehran, Iran.*; b *School of Pharmacy, Shahid Beheshti University of Medical Sciences, Tehran, Iran.*; c * Student Research Committee, Shahid Beheshti University of Medical Sciences, Tehran, Iran.*; d *Department of Microbiology, Faculty of Basic Sciences, Islamic Azad University, North Tehran Branch, Tehran, Iran.*

**Keywords:** Iranian black tea, *Camellia sinensis*, *Echium amoenum*, Cowslip flower, Tumor Necrosis Factor-alpha (TNF-alpha)

## Abstract

Many species of tea (*Camellia sinensis*) and cowslip (*Echium amoenum*) are used in Iranian traditional medicine. The aim of this study was to conduct the survey on the ability of Iranian black tea and cowslip extracts on secretion of tumor necrosis factor-alpha (TNF-alpha) by non-infected and infected mouse macrophages. A macrophage infection model with *Legionella pneumophila *and enzyme linked immunosorbent assay (ELISA) technique was used in this study. Research showed that the concentrations of TNF-alpha in non-infected and infected macrophage culture supernatant treated with various concentrations of Iranian black tea and cowslip extracts was significantly higher than the control. Various concentrations of cowslip (0.5, 5 and 50 μg/mL) had significantly a great effect on the induction of TNF-alpha secretion in comparison with the Iranian black tea extract (*P *< 0.0001). In conclusion, we demonstrated the ability of Iranian black tea and cowslip on the induction of TNF-alpha, which can exert an anti-*L. pneumophila *activity on macrophages at a low dose. However, further studies to elucidate the mechanism(s) of the induction of macrophages by Iranian black tea and cowslip as well as their potential inhibitory effects on the growth of infected cells and their possible antitumor effects should be carried out in future works.

## Introduction

Many species of cowslip (*Echium amoenum*) and tea (*Camellia sinensis*) are used in Iranian traditional medicine. Cowslip grows in many geographical regions of Iran. This plant contains mucilage, carbohydrates, flavonoids, anthocyanin, Na^+^ and K^+^ ions, tannins, phenols, and some alkaloids that have tranquilizer, anti-anxiety and diuretic effects. Tea has been known traditionally as a healthy drink with some beneficial effects on health. Its effects on health had not been demonstrated by well-controlled laboratory studies until the 1970s (1). However, current studies have revealed the biological effects of tea, such as antitumor and antimicrobial effects, even at the molecular level.

The active components of tea responsible for such biological effects are now known to be catechins (also known as polyphenols), which constitute seven forms, including epigallocatechin gallate (EGCg). EGCg is a major catechin compound in tea extract and is also the most active form among the tea catechins in a variety of biological activities (2). For instance, EGCg has anticarcinogenic (3), antioxidant (3, 4), as well as antimicrobial activities (5-8). Although the mechanism of antimicrobial activity of EGCg has been studied (5), it is still unclear. The immunomodulatory effect of EGCg has been increasingly recognized (9, 10) and it is known that EGCg potently stimulates the production of interleukin-1-alpha (IL-1-alpha), IL-1-beta, and tumor necrosis factor-alpha (TNF-alpha) by cultured human peripheral blood mononuclear cells (11). Furthermore, EGCg protects against ultraviolet (UV) radiation-induced immunosuppression and tolerance induction by reducing IL-10 production and increasing IL-12 production in epidermal and dermal cells (12). However, the detailed immunomodulatory effects of EGCg have not been investigated. 

In this study, a macrophage infection model with *L. pneumophila *(13) was used to determine the ability of Iranian black tea and cowslip extract to induce of TNF-alpha secretion from macrophages. 

## Experimental

**Materials**

Plant samples were collected from North region of Iran. Iranian black tea was collected from Hassansara, Roodsar district, Gilan Province in 2006. Cowslip was collected from Khalkhal Mountains, Ardabil Province in 2007, dried in room temperature and identified in, School of Pharmacy, Shahid Beheshti University of Medical Sciences, Tehran, Iran. 

The decoction method was used for preparing aqueous solution of the herbal extracts. For this purpose 100 g of dried plant powder (Iranian black tea or cowslip) was dissolved in 1 L of distilled water and the mixture decocted for 5 min. After filtering, the extract was dried using water bath. Each 100 g of dried plant yielded about 15 g of dried extract powder (14, 15). The different concentrations of extracts were prepared using dried extract powders and distilled water as the solvent . These concentrations were 0 (as blank or control group), 0.5, 5, and 50 μg/mL).

**Methods**

*Macrophage cell culture*

The J774 A.1 murine (BALB/c Mouse) macrophage cell line, purchased from the cell bank of Pasteur Institute of Iran, was used in this study. The cells were maintained in RPMI 1640 medium (Biosera, Spain) containing 10% heat-inactivated fetal calf serum (FCS, Baharafshan, Iran). The J774 A.1 cells were allowed to adhere to 24-well tissue culture plates at a concentration of 5×10^5^ cells/mL for 2h in 5% CO_2_ at 37°C. The resulting monolayer cells were washed with Hanks’ balanced salt solution (Baharafshan, Iran), supplied with 10% FCS-RPMI 1640 medium without antibiotics, and then used for experiments (16).

*Bacteria*

*L. pneumophila *M138 was obtained as a gift from Dr. Mojtaba Moosavian (Department of Microbiology, School of Medicine, Ahvaz Jundishapur University of Medical Sciences, Ahvaz, Iran). The bacteria were cultured on BCYE Medium (Sigma) for 3 days at 37°C. The bacterial suspensions were prepared in pyrogen-free saline, and the concentrations of bacteria were determined by spectrophotometry (17).

*Macrophage infection*

The macrophage monolayers were infected with *L. pneumophila *(infectivity ratio of 10 bacteria per cell) for 30 min, washed to remove non-phagocytized bacteria, and incubated in RPMI 1640 medium containing 10% FCS with no antibiotics. The cultures were then incubated for up to 48 h at 37 °C in 5% CO_2_ (13).

*Macrophage cell culture treatment with extracts*

The macrophage cultures (non-infected and infected with bacteria) were treated with various concentrations (0, 0.5, 5, and 50 μg/mL) of Iranian black tea and cowslip extracts for up to 24 h at 37°C in 5% CO_2_.

*ELISA technique*

The concentrations of TNF-alpha in the culture supernatants of macrophage cultures were determined by Sandwich ELISA, using matched antibody pairs for ELISA (ELISA washer and microplate reader: Anthos 2020, Austria). Various concentrations were calculated, using the standard curve constructed for each plate.

*Statistical analysis*

For performing descriptive statistics on raw data and statistical analyses, independent sample T-test and Tukey post-test were used (SPSS software, Version 13). Statistical significance was taken at *P *≤ 0.05.

## Results and Discussion

*Direct effect of iranian black tea extract on TNF-alpha production by non-infected macrophages*

Compared to the control group, various concentrations of Iranian black tea extract (0.5, 5 and 50 μg/mL) significantly induced secretion of TNF-alpha from non-infected macrophages (*P*<0.0001). The data showed statistically significant differences between the effect of different concentrations of cowslip extract on the production of TNF-alpha.

*Direct effect of iranian black bea extract on TNF-alpha production by infected macrophages*

Compared to the control group, various concentrations of Iranian black tea extract (0.5, 5 and 50 μg/mL) significantly induced secretion of TNF-alpha from the infected macrophages (*P*<0.0001). The data obtained from this study showed no statistically significant difference between the effect of 5 and 50 μg/mL concentrations on the production of TNF-alpha (*P *< 0.05).

*Direct effect of cowslip extract on TNF-alpha production by non-infected macrophages*

Compared to the control group, various concentrations of cowslip (0.5, 5 and 50 μg/mL) significantly induced secretion of TNF-alpha from non-infected macrophages (*P *< 0.0001). The data showed statistically significant differences between the effect of different concentrations of cowslip extract on the production of TNF-alpha (i.e., with increasing the concentration of cowslip extract, the concentration of TNF-alpha also increased) (*P *< 0.001).

*Direct effect of cowslip extract on TNF-alpha production by infected macrophages*

 Compared to control group, various concentrations of cowslip (0.5, 5 and 50 μg/mL) significantly induced secretion of TNF-alpha from infected macrophages (*P *< 0.0001). The data showed statistically significant differences between the effect of different concentrations of cowslip extract on the production of TNF-alpha (i.e., increasing the concentration of cowslip extract leads to an augmentation of the concentration of TNF-alpha) (*P *< 0.001).

Briefly, impact of various concentrations of Iranian black tea and cowslip present within non-infected and infected groups on the induction of TNF-alpha were statistically significant (*P *<0.0001). In other words, these two extracts induced production of TNF-alpha compared to the control groups.

*Comparson of the difference between the effect of iranian black tea and cowslip extracts on TNF-alpha production by non-infected macrophages*

 Various concentrations of cowslip (0.5, 5 and 50 μg/mL) had a significantly greater effect on the induction of TNF-alpha secretion in comparison with the Iranian black tea extract (*P*< 0.0001). 

*Comparing the difference between the effect of iranian black tea and cowslip extracts on TNF-alpha production by infected macrophages*

 There were significant differences between the of 0.5 and 5 μg/mL (but not 50 μg/mL) concentrations of cowslip and Iranian black tea on induction of TNF-alpha secretion. In other words, compared to the Iranian black tea extract, the cowslip extract had a greater effect on the production of TNF-alpha (*P *< 0.0001). 

**Figure 1:**
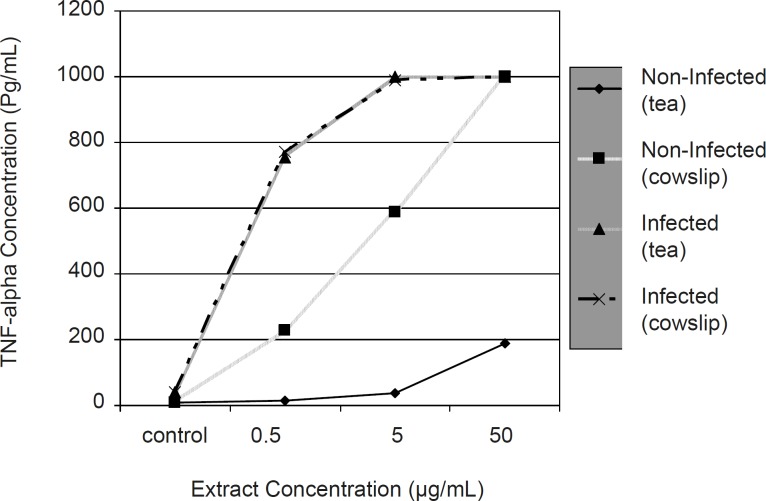
Comparison of the effect of Iranian black tea and cowslip extracts (with different concentrations) on TNF-alpha production by non-infected and infected macrophages during 24 h incubation period

Figure 1 compares of the effect of Iranian black tea and cowslip extracts (with different concentrations) on TNF-alpha production by non-infected and infected macrophages, during 24 h incubation period.

In this study, we showed the potent effects of Iranian black tea and cowslip extracts on the induction of TNF-alpha production from infected and non-infected macrophages, even at a concentration as low as 0.5 μg/mL, during a period of 24 h incubation time. These data suggest that the test extracts can result in special effects on the activity of macrophage, a pivotal element of immune system that is the main cell in killing bacteria as well as acting against tumor cells. 

It has been reported that EGCg itself induces TNF-alpha from peripheral blood mononuclear cells (11). It can be suggested that TNF-alpha may be involved in the anti-*L. pneumophila *activity of macrophages induced by the Iranian black tea and cowslip extracts. However, the direct effect of these extracts on the growth of *L. pneumophila *should be assayed directly. 

As TNF-alpha is one of the most important cytokines secreted from immune cells during the defense process against microbes and tumor cells, it can be conjectured. Therefore, our data propose a potential role for Iranian black tea, and particularly cowslip, in the defense process against infections and tumor cells. Previous studies on green tea have shown bactericidal effects of catechin components of green tea (5-7) and cowslip (18). In addition, it has been reported that the inflammatory cytokine TNF-alpha is required for the prompt resolution of pneumonic legionellosis and points to a direct role for TNF-alpha in the activation of phagocytes (19). Also, existing evidences have shown the cancer chemopreventive effects of green tea in several animal tumor models (3, 20-22). Conversely, Cao et al. have shown green tea decreases pro-inflammatory tumor necrosis factor mRNA levels in rat (23). This discrepancy may be due to the different environments used, as well as the different exposure time of animal cells to extracts.

In conclusion, in this study we demonstrated the ability of Iranian black tea and cowslip to induce TNF-alpha, which can exert an anti-*L. pneumophila *activity on macrophages at a low dose. Further studies to elucidate the mechanism(s) of the induction of macrophages by Iranian black tea and cowslip, their potential inhibitory effects on the growth of infected cells and their possible antitumor effects should be carried over in the future.
